# Changing the Tolerance of the Intolerant: Does Large Carnivore Policy Matter?

**DOI:** 10.3390/ani14162358

**Published:** 2024-08-15

**Authors:** Erik R. Olson, Jamie Goethlich

**Affiliations:** 1 Department of Natural Resources, Northland College, Ashland, WI 54806, USA; 2 Department of Forest and Wildlife Ecology, University of Wisconsin-Madison, Madison, WI 53706, USA; goethlich@wisc.edu

**Keywords:** *Canis lupus*, carnivore conservation, lethal control, policy, tolerance, wildlife management, Wisconsin, wolf

## Abstract

**Simple Summary:**

How people feel about large carnivores can be critical in determining the success of conservation efforts. In some cases, people’s attitudes towards large carnivores are more influenced by policies dictating how people can interact with those species rather than the species themselves. Yet, the connections between policy and tolerance of large carnivores remain unclear. To better understand these connections, we surveyed residents of northern Wisconsin, USA, about wolves *Canis lupus* and wolf policies. We grouped survey respondents based on their general attitudes towards wolves and assessed whether respondents expected their tolerance of wolves to improve under different policy scenarios. Hunters, people with generally negative or ambivalent attitudes towards wolves, and people with wildlife conflict experience were more likely to expect their tolerance to improve under policy scenarios that allowed for the regulated killing of wolves under certain circumstances. However, we also observed important nuances in the relationship between tolerance of wolves and wolf policy. Large carnivore conservationists must balance conservation objectives with the preferences of local people. The fulcrum of this balance may shift over time as local preferences or species status change. Thus, monitoring local policy preferences may be just as important as carnivore population monitoring.

**Abstract:**

Success in large carnivore conservation often hinges on local residents’ tolerance towards those species. Feelings of powerlessness and frustration with wildlife policies can lead to intolerance of the species. In extreme cases, intolerance may manifest in poaching. Thus, changes in policy may influence the tolerance of wildlife. To examine the connections between policy and tolerance, we examined how policy scenarios influenced anticipated changes in tolerance to wolves *Canis lupus*. We administered a survey in 2015–2016 in the core wolf range within northern Wisconsin, USA. Using hierarchical cluster analysis, we clustered respondents into groups based on their current tolerance of wolves. We evaluated the behavioral intentions of the clusters and examined the influence of policy scenarios on respondents’ anticipated changes in tolerance. Finally, using an information-theoretic model selection framework, we assessed the effects of tolerance clusters and demographic factors. The respondents were clustered into three clusters relative to their current tolerance towards wolves: positive, ambivalent, and negative. Each cluster exhibited significantly different behavioral intentions and anticipated changes in tolerance for all scenarios. In all scenarios, respondents who already held positive attitudes towards wolves were significantly less likely to report expected changes in tolerance toward wolves following changes in wolf management. However, respondents who held ambivalent or negative attitudes towards wolves were significantly more likely to report expected changes in tolerance towards wolves following changes in wolf management. Regarding a regulated wolf hunting and trapping season, we observed a Simpson’s Paradox, wherein, when examined in aggregate, no clear pattern emerged, but when examined at the cluster level, important and intuitive patterns emerged. Our demographic model results suggest that policy changes resulting in greater state management authority over wolves, especially authority to implement certain forms of legal killing of wolves, could result in significant increases in tolerance for individuals who identify as hunters, have lost livestock to a predator, or are currently ambivalent or negative towards wolves. Our work elucidates the nuanced relationship between tolerance of wildlife and wildlife policy and identifies a potential ecological fallacy.

## 1. Introduction

Tolerance towards wildlife can determine whether conservation efforts succeed or fail [1,2,3]. Positive attitudes towards wildlife can lead to greater tolerance, creating a social environment receptive to wildlife conservation (e.g., refs. [1,4,5]). On the other hand, intolerance towards wildlife can challenge conservation efforts (e.g., refs. [1,6]). For species that create real or perceived conflicts with humans, such as large predators, local tolerance is especially critical [1]. Conflict with wildlife can lead to greater intolerance and, in some cases, enhanced sociopolitical conflict over the best ways to achieve both conservation and local livelihood interests [7,8,9,10,11,12,13,14]. To maintain populations of conflict-associated species, management agencies often rely on strategies designed to enhance local tolerance for the species or, perhaps more directly, acceptance of how the species is being managed (e.g., ref. [7]). Such policies have been developed and implemented globally with varying degrees of success (e.g., refs. [12,15,16]). Feelings of powerlessness, anger, and frustration with existing or inconsistent wildlife policies can also lead to intolerance towards wildlife [1,9,11,14,17,18,19]. In extreme cases, such frustrations may manifest into behaviors that negatively affect wildlife, such as poaching, retaliatory killing, and other expressions of intolerance [9,11,13,14,20,21].

In instances where a species is viewed negatively, some researchers, conservationists, and policymakers have suggested that changes in policy or management strategy may lead to greater tolerance or acceptance of wildlife [14,22]. In some cases, decisions have been made without formal assessment of anticipated changes in tolerance as a result of a policy or management decision. In other cases, researchers have used social science to examine public acceptance of wildlife policies to ensure wildlife policy is compatible with stakeholders’ attitudes (e.g., refs. [23,24]). Social scientists have also evaluated changes in tolerance as they relate to past changes in wildlife management via the use of longitudinal attitude surveys (e.g., refs. [2,18]). These approaches can be used to better understand how societies relate to various wildlife policies and to guide wildlife decision-making. 

### 1.1. Measuring Tolerance 

Tolerance, as a concept, has been plagued, to a degree, with ambiguity [6,25,26,27]. Moreover, wildlife researchers have defined and measured tolerance in a variety of ways, which has added further complexity. Bruskotter and Fulton [6] portrayed tolerance as part of a gradient: intolerance–tolerance–stewardship (see Figure 1 in ref. [28]). Bruskotter and Fulton [6] considered tolerance to be synonymous with acceptance; a passive concept requiring no action. They defined intolerance and stewardship as behaviors that may negatively or positively impact wildlife, respectively [6] (though see ref. [25]). St. John et al. [28] largely agree but went further to broaden the idea of tolerance by saying that it “is not *necessarily* expressed through tangible acts” (p. 2, emphasis added; see also ref. [25]). Thus, the role of passivity was established along the tolerance gradient and the blurred lines were acknowledged between the concepts of intolerance, tolerance, and stewardship. Others have also drawn connections between the ideas of tolerance and coexistence [1,29]. For example, Expósito-Granados et al. [30], influenced by refs. [1,29], defined tolerance “…as the ‘human acceptance’ of the risks and damages caused by carnivores, a necessary condition to achieve a permanent coexistence”. Thus, tolerance as a concept remains critically relevant but also somewhat ambiguous.

Given that tolerance can be expressed through action or inaction, some researchers have used poaching as a metric of tolerance and have assessed how changes in policy may correspond to changes in poaching—i.e., tolerance (e.g., refs. [11,31]). In other cases, attitudes and behavioral intentions are often used as measures of tolerance (e.g., refs. [18,26,32,33]), and researchers have demonstrated that tolerance for wolves *Canis lupus* can be quantified via attitude surveys [25,26]. Bruskotter et al. [26] evaluated how well attitudes, associated with tolerance, correlated with behavioral measures and concluded that “…attitudinal measures represent valid, parsimonious measures of tolerance that may be useful when behavioral measures are too cumbersome or misreporting of behavior is anticipated” (p. 225). Hazzah et al. [34], concurred, finding that “the strongest predictor of lion-killing behavior was attitudes towards lions” [12]. Moreover, controversial species, such as large predators, often produce stronger relationships between attitudes and behaviors (refs. [35,36,37,38], as cited in ref. [26]). Thus, attitude surveys may enhance our understanding of how local tolerance changes as a result of changes in policy and provide important insights into conservation decision-making.

### 1.2. Wolf Conservation as a Case Study

As a large carnivore species distributed throughout the Northern Hemisphere, wolves come into direct conflict with humans, attacking and occasionally killing domestic animals, livestock, and, on rare occasions, humans [39,40,41,42]. Humans may also perceive wolves as competitors for culturally or economically important wild ungulates [18,43]. Human–human conflict over wolf management is also prevalent [8,11,44]. Thus, tolerance of wolves is an important topic of consideration for conservationists and policymakers across the Northern Hemisphere. 

Research regarding the effects of policy changes on wolf tolerance has produced two mutually exclusive hypotheses. First, the empowerment or killing for tolerance hypothesis predicts policies that reduce protections for wolves will result in increased tolerance of wolves [22]. For example, Olson et al. [11] found that annual mortality rates attributed to the illegal killing of wolves in Wisconsin decreased during times when the state had management authority to kill wolves that attacked or killed livestock or pets near homes. Additionally, they argued that frustrations associated with inconsistency in management authority may have led to declining tolerance towards wolves and a legislatively mandated wolf hunt (see also refs. [18,20,21,44]). Likewise, Liberg et al. [45] found that poaching, i.e., intolerance, appeared to decrease during periods with more aggressive wolf policies (though see refs. [43,46,47,48,49]). A second hypothesis, the devaluing-policy-signal hypothesis, predicts that policies that reduce protections for wolves send a signal lowering the value of wolves to the public and result in an increase in intolerance towards wolves [31]. Chapron and Treves [31] found wolf population growth in Wisconsin slowed during times where the state had management authority to kill wolves and suggested that such decreases in growth were associated with cryptic poaching. However, Pepin et al., [50], Olson et al. [51], and Stien [52] all pointed to flaws in that work. Using the same data as Chapron and Treves [31], Stein [52] found the opposite conclusion—allowing wolf culling was more likely to decrease poaching than increase it, when accounting for the effect of culling on reproductive rates. However, Santiago-Avila et al. [53] reported that their work provided additional support for the devaluing-policy-signal hypothesis, stating that intolerance (i.e., poaching) increased during times with more aggressive forms of wolf management. Although their analysis relied mainly on collared wolves that went missing instead of confirmed poaching incidents. Clearly, the scientific debate regarding the effects of policy changes on public tolerance for wolves has not yet been resolved. Nevertheless, policy changes are proposed, hotly debated, and occur frequently [11,54]. 

Building upon this important area of inquiry, we assessed tolerance for wolves among residents within the core wolf range in Wisconsin, USA. We evaluated respondents’ anticipated changes in tolerance for wolves under three different policy or management scenarios. We hypothesized respondents’ attitudes toward wolves would occur along a gradient, but would generally fit into three categories, those who always viewed wolves positively, those who always viewed wolves negatively, and those who held more moderate views. Furthermore, following the empowerment hypothesis, we expected respondents with more negative attitudes towards wolves would indicate greater anticipated increases in tolerance for all three scenarios than those that were ambivalent and positive towards wolves, respectively. At the time the survey was distributed, wolves had been recently federally protected under the Endangered Species Act in the state of Wisconsin, following three consecutive years with wolf hunting seasons in the state.

## 2. Materials and Methods

We measured public attitudes toward wolves using a subset of questions from a larger, six-page survey of attitudes toward wolves and other carnivores [55]. We designed the survey to be confidential and anonymous and informed potential respondents they could skip any questions they did not feel comfortable answering and that they may end the survey at any time. We also informed potential respondents that we would summarize and publicly distribute survey results, and we required that all respondents be 18 years of age or older. For detailed information regarding response rates, respondent demographics, willingness-to-participate, and non-response bias, see ref. [55].

We distributed the survey as reported in ref. [55], hand-delivered at local convenience and grocery stores. We limited the possibility of re-sampling individuals by limiting the number of survey periods and surveys per sampling location relative to their assumed customer base. Additionally, we avoided re-sampling by surveying establishments at different times of day and days of the week to avoid re-sampling regular patrons, and we asked individuals whether they had already taken the survey. We used a quasi-systematic sampling strategy to identify potential respondents. We approached every third person [56]. If the third person was not willing to take the survey, we continued approaching every subsequent person until a respondent took the survey. We followed a survey script, and we gave each respondent the option of completing the questionnaire in-person or mailing it back. We administered the survey from November through January in 2015–2016. We entered data into a database and quality-assured the entire database to ensure each survey was entered correctly. We restricted all analyses to respondents who self-identified as Wisconsin residents ≥18 years-of-age. Our protocol was reviewed and approved by the Northland College Institutional Review Board (NC IRB protocol #101).

### Data Analysis 

**Figure 1:**
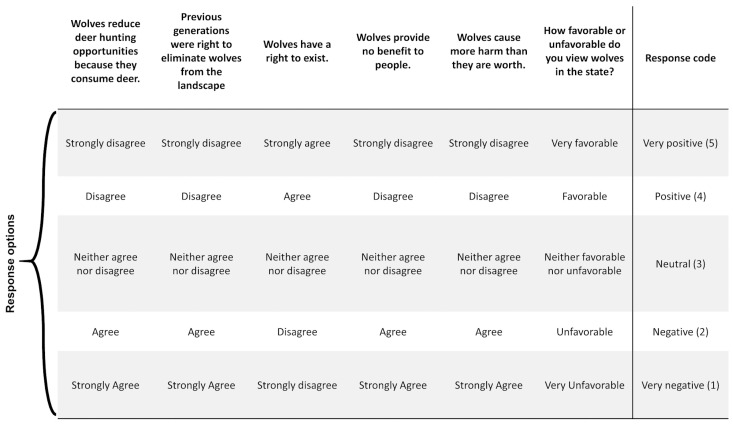
Six survey questions used in cluster analysis and their associated response choices. For analyses, we reconfigured responses for these six questions into the corresponding scoring system depicted in the column on the far right side of the figure.

We asked six questions to create a composite index of respondents’ overall attitudes toward wolves. Bruskotter et al. [26] found that the use of composite attitude indices correlated well with behavioral measures and was therefore an appropriate approach for assessing tolerance [57]. For analyses, we coded responses on a scale from 1 to 5 where 1 was “very negative” and 5 “was very positive” (Figure 1). We then used cluster analysis to assess whether respondents could be generalized into groups based on their responses to six questions about wolves outlined in Figure 1. Such analysis would partition individual respondents into clusters with varying degrees of tolerance towards wolves [26,57]. To conduct the cluster analysis, we first explored various clustering combinations using the function *fviz_nbclust* in the R package *factoextra* Version 1.0.7 [58]. We then used the elbow method to determine the optimal number of clusters in the data. The elbow method is a technique used to visually determine an inflection point (elbow) in a dataset by applying a K-means clustering algorithm to the dataset and comparing the within-cluster sum of squares at a range of K values. Using this technique, we determined the inflection point in our dataset occurred at three clusters. We then partitioned respondents into three clusters using complete-link agglomerative hierarchical cluster analysis using the *hcut* function in *factoextra* [58]. For reference purposes, we named clusters based on the prevailing attitudes of most respondents in each respective cluster. 

To validate our tolerance clusters, we assessed the behavioral intentions of each cluster using responses to the following question, “Let’s imagine you have $100 specifically for wolf management, how much of that $100 would you put towards each of the following wolf management options? *You can give all of the money to one option or you can split it up across multiple options. Should sum up to $100*”. With the following options: “Delist wolves from the endangered status and allow management authority to return to the state”, “Keep wolves federally protected as an endangered species under the Endangered Species Act”, “Reclassify wolves as a threatened species under the Endangered Species Act, which would keep wolves federally protected while providing the state with management authority to kill wolves that attack livestock or pets near homes”, or “Eliminate wolves from the state”. We plotted the mean and standard error of the USD amount respondents would give to support efforts to achieve each of the four different policy outcomes for wolves in Wisconsin. Based on the strong association between attitudes and behaviors or behavioral intentions detected by others, we expected greater mean values for options that aligned with a particular tolerance cluster. For example, we expected higher mean values for the “delist” and “eliminate” options for the negative cluster, higher mean values for the “keep” option for the positive cluster, and higher mean values for the “reclassify” option for the ambivalent cluster. We used one-way ANOVA to test for differences among clusters and post hoc Tukey’s test of contrasts to determine whether clusters differed significantly from one another. We used α = 0.05 to determine significance. 

We analyzed the anticipated change in tolerance for all respondents combined and for each cluster separately. To assess anticipated change in tolerance in response to changes in wolf management, we examined the aggregate and cluster-level responses to the following scenarios: “My tolerance for Wisconsin wolves would increase if…”: (1) “…they were not federally protected”, (2) “…the state had management authority to kill wolves attacking livestock or pets near homes”, (3) “…there was a regulated hunting and trapping season for wolves”. We used one-way ANOVA to test differences among clusters, and post hoc Tukey’s test to determine whether clusters differed significantly from one another across all scenarios. We used α = 0.05 to determine significance. 

We tested the effect of respondent cluster assignment and demographic variables on respondents’ anticipated change in tolerance. Based on previous research, we created a suite of 20 generalized linear models to explore factors that influence respondents’ anticipated change in tolerance under each management scenario. Models included demographic variables (i.e., gender, age, and education), life experience variables (i.e., whether they owned livestock, experience with the loss of livestock to wildlife, and whether individuals identified as hunters), and opinions toward wolves (i.e., tolerance cluster). We determined whether clusters differed significantly from one another by conducting a post hoc Sidak on the top model for each management scenario. We conducted all analyses using program R version 4.1.2.

## 3. Results

**Figure 2:**
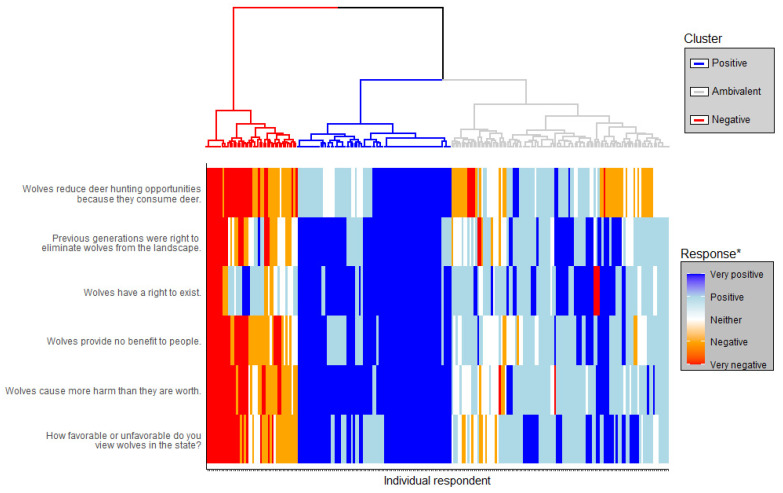
Tree diagram depicting clusters of respondents created using complete-link hierarchical cluster analysis. Colors correspond to the clusters (red = Negative, blue = Positive, gray = Ambivalent). Each respondent in the tree diagram is aligned with their specific responses to the six questions we used in the cluster analysis. Figure created by Jamie Goethlich with input from Erik R. Olson.

Of the 984 potential respondents contacted, 594 agreed to participate in the survey. Of those, we received 276 completed surveys. After removing respondents who were not Wisconsin residents and those who later reported to be <18 years of age (every potential respondent was asked if they were ≥18 years-of-age prior to receiving the survey), we were left with 232 respondents included in our analyses. Our complete-link hierarchical clustering analysis placed respondents into three clusters of 78, 108, and 46 respondents (Figure 2, Table S1). For discussion purposes, we named these clusters *Positive*, *Ambivalent*, and *Negative*, respectively, based on the prevailing attitudes of each cluster (Figure 2).

**Figure 3:**
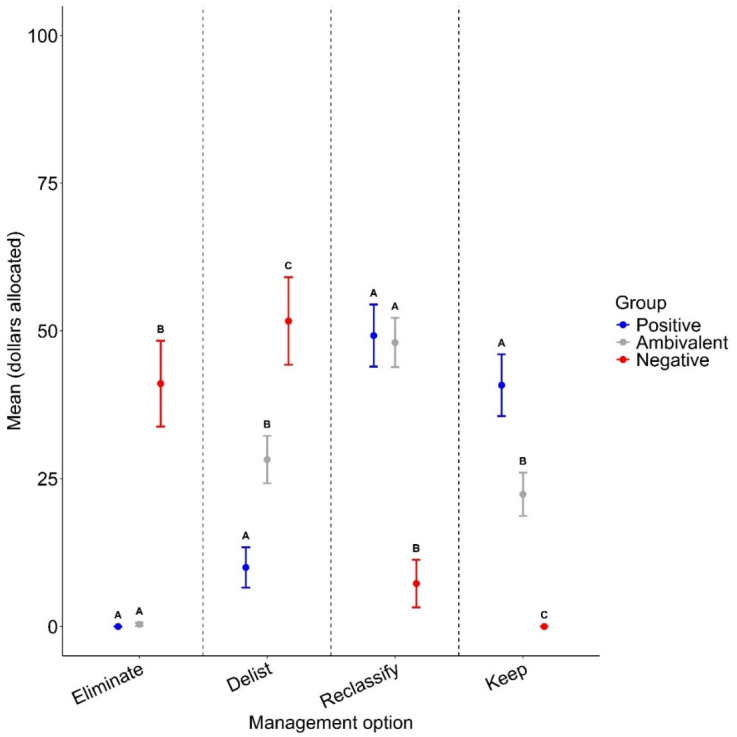
Mean (±standard error) USD hypothetically allocated by respondents to each of the following wolf policy options according to tolerance cluster group (colors). Respondents’ behavioral intentions were assessed using the following question: “Let’s imagine you have $100 specifically for wolf management, how much of that $100 would you put towards each of the following wolf management options? *You can give all of the money to one option or you can split it up across multiple options. Should sum up to $100*”. with the following options: “Delist wolves from the endangered status and allow management authority to return to the state”, “Keep wolves federally protected as an endangered species under the Endangered Species Act”, “Reclassify wolves as a threatened species under the Endangered Species Act, which would keep wolves federally protected while providing the state with management authority to kill wolves that attack livestock or pets near homes”, or “Eliminate wolves from the state”. Tukey letters above confidence intervals indicate significant differences (at α = 0.05) between cluster means.

Behavioral intentions of each cluster were as expected, with the *Positive* cluster indicating an intention to financially support efforts to keep wolves federally endangered or reclassify wolves as threatened under the Endangered Species Act, the *Negative* cluster indicating an intention to financially support efforts to eliminate wolves from the state or delist wolves from the Endangered Species Act, and the *Ambivalent* cluster indicating intention to financially support efforts to delist wolves from the Endangered Species Act, keep wolves federally endangered, or reclassify wolves as threatened under the Endangered Species Act (Figure 3). One-way ANOVA indicated statistically significant differences among clusters for each money allocation scenario (eliminate scenario, *F* = 63.1, *df* = 206, *p* < 0.001; delist scenario, *F* = 15.9, *df* = 206, *p* < 0.001; reclassify scenario, *F* = 18.1, *df* = 206, *p* < 0.001; keep scenario, *F* = 17.8, *df* = 206, *p* < 0.001; see Tukey’s post hoc test results in Figure 3).

**Figure 4:**
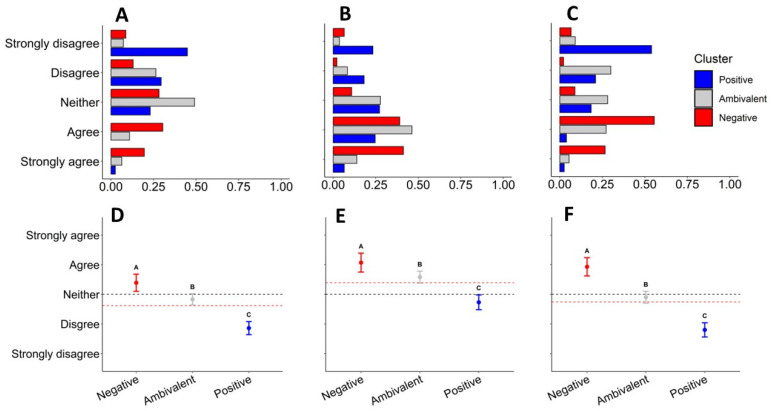
(**A**,**D**) represent responses to the scenarios where wolves were not federally protected, (**B**,**E**) represent responses to the scenario where the State of Wisconsin had management authority, and (**C**,**F**) represent responses to the scenario where there was a regulated wolf hunting season. Panels in the top row depict the proportion of respondents by cluster. Panels in the bottom row depict the level of anticipated increase in tolerance (±95% *CI*) for the Positive, Ambivalent, and Negative clusters. Tukey letters above confidence intervals indicate significant differences (at α = 0.05) between means. The black dashed line indicates a neutral response, and the red dashed line indicates the overall average response for each question.

To compare how tolerance of wolves for each cluster might change under different wolf management policies, we compared the responses to three plausible wolf management scenarios. The three scenarios included removing federal protections for wolves, giving the State of Wisconsin management authority to kill wolves attacking livestock or pets near homes, and implementing a wolf hunting season. One-way ANOVA indicated statistically significant differences among clusters for all three management scenarios (*p* < 0.05; Figure 4; “not federally protected”, *F* = 38.44, *p* < 0.05; “regulated hunting season”, *F* = 59.82, *p* < 0.05; “state management authority”, *F* = 24.62, *p* < 0.05). Additionally, we ran a Tukey’s post hoc test to compare responses among the *Positive*, *Ambivalent*, and *Negative* clusters for each management scenario. All three clusters were significantly different from one another under all three policy scenarios (*p* < 0.05; Figure 4). In all three policy scenarios, the *Positive* cluster was the least likely to indicate that their tolerance for wolves would increase (Figure 4). Alternatively, the *Negative* cluster was more likely to indicate their tolerance for wolves would increase for all three management scenarios (Figure 4). When converted to a five-point scale where 1 corresponds to Strongly Disagree and 5 corresponds to Strongly Agree, the scenario where the state was given management authority yielded a mean value of 3.39, indicating a slight positive relationship between state management authority and overall anticipated changes in wolf tolerance (Figure 4). Overall, respondents were more likely to report that their tolerance would not improve under a scenario where wolves lost federal protection (mean = 2.62; Figure 4). The difference in average agreement between *Positive* and *Negative* clusters was most extreme in the scenario where wolves were hunted, with a difference of 2.13, compared to a difference of 1.53 for the “not federally protected” scenario and 1.34 under the “state management authority” scenario (Figure 4).

**Figure 5:**
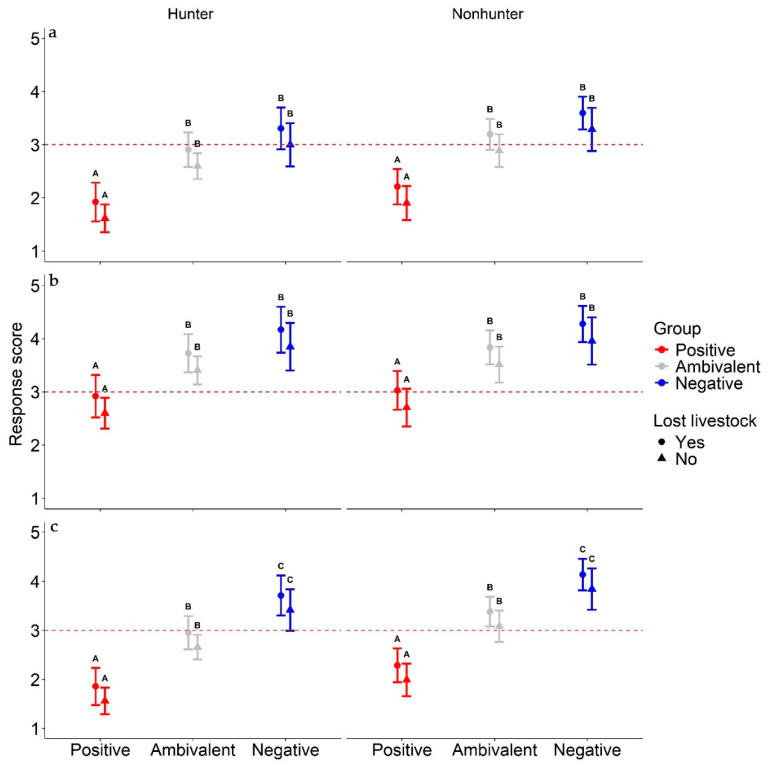
Predicted anticipated change in tolerance towards wolves (1–5, 1 = very unlikely to change, 5 = very likely to change) under three different policy change scenarios: "My tolerance for Wisconsin wolves would increase if…”: (a) “…they were not federally protected”, (b) “…the state had management authority to kill wolves attacking livestock or pets near homes”, (c) “…there was a regulated hunting and trapping season for wolves”, based on the results of a generalized logistic regression model of human attitude data completed in northern Wisconsin, USA, 2015 (*n* = 212). Predicted response varied by hunter identity, experience with livestock loss to predators, and tolerance assignment cluster (i.e., initial tolerance towards wolves). Sidak letters above confidence intervals indicate significant differences (at *α* = 0.05) between cluster means.

Generalized linear regression model comparison identified that hunter identity, loss of livestock to wildlife, and tolerance cluster assignment were the most important parameters affecting respondents’ anticipated change in tolerance towards wolves as it related to the three policy change scenarios (Table 1). The top models for both “…not federally protected” and “…regulated hunting season” scenarios included hunter identity (*Hunter*), loss of livestock to wildlife (*Lost Livestock*), and tolerance cluster assignment (*Cluster*) (*ω_i_* = 0.46, for both; Tables S1 and S3). The top model for the “…state had management authority to kill wolves attacking livestock or pets near homes” scenario included loss of livestock to wildlife and tolerance cluster assignment (*ω_i_* = 0.45), and the second highest ranked model (Δ*AIC_c_* = 1.68) contained hunter identity, loss of livestock to wildlife, and tolerance cluster assignment (*ω_i_* = 0.19; Table S2). Regardless of the scenario, respondents identifying as hunters or those who had experience with the loss of livestock to wildlife were more likely to anticipate an increase in tolerance, with a change in wolf management policy (Table 1; Figure 5). Individuals who were ambivalent or negative towards wolves (i.e., assigned to ambivalent or negative initial tolerance clusters) were also more likely to anticipate increased tolerance towards wolves for all three management scenarios (*β* min and max including 95% *CI* = 0.48–2.28; Table 1), while those that were positive towards wolves (i.e., positive tolerance cluster) were less likely to anticipate increases in tolerance (Table 1; Figure 5). 

## 4. Discussion

Our survey results suggest people living in Wisconsin’s wolf range likely fall into one of three clusters regarding their attitudes toward wolves: *Positive*, *Negative*, and *Ambivalent*, characterized by their prevailing attitudes towards wolves. These results were further validated by the behavioral intentions of each cluster (Figure 3 and Figure 4), which suggests that individuals from these clusters hold behavioral intentions that correspond with these general attitudes—further affirming the connections between attitudes and behaviors or behavioral intentions found by others [26,34]. We also found that individuals less tolerant or more ambivalent towards wolves were more likely to report an anticipated increase in tolerance if policy changed from protected as an endangered species to state management authority or legal lethal removal of wolves (lethal control or regulated hunting and trapping). Unsurprisingly, respondents who already held positive attitudes towards wolves were significantly less likely to report anticipated changes in tolerance toward wolves following changes in wolf management that led to an increase in the legal killing of wolves. Regarding all policy scenarios, but particularly a regulated wolf hunting and trapping season, we observed a Simpson’s Paradox, wherein, when examined in aggregate, no clear pattern emerged, but when examined at the cluster level, important and intuitive patterns emerged. Similarly, Naughton-Treves et al. [59] demonstrated that social organization was the strongest predictor of attitudes towards wolves and wolf management. Bradshaw et al. [19] also found significant differences in attitudes towards wolves across social groups. Clearly, such findings encourage caution when interpreting aggregate data without considering more nuanced patterns at different levels of social organization. 

Our results also highlight that nuance plays an important role in shaping tolerance toward wolves. Accordingly, focus group studies or targeted surveys designed to better understand factors shaping the tolerance of intolerant (or ambivalent) social groups [17,59] may elucidate further important pathways to cooperation among stakeholders. Beardmore [57] used a similar clustering approach to identify areas of common ground and points of contention for the most vocal of stakeholders regarding wolf management in Wisconsin. Comparing the attitudes of vocal stakeholder groups to the attitudes of the general public can further provide valuable insights for state management agencies [19]. 

### 4.1. Factors Influencing Anticipated Changes in Tolerance

Our examination of demographic variables associated with anticipated increases in tolerance under three policy scenarios resulted in a common (all three scenarios) model that included loss of livestock to a predator (positive effect), identity as a hunter (positive effect), and tolerance cluster (ambivalent and negative clusters had positive effects). Our model results suggest that policy changes resulting in greater state management authority over wolves, especially authority to implement certain forms of legal killing of wolves, could result in increases in tolerance for individuals who identify as hunters, have lost livestock to wildlife, or are currently ambivalent or negative towards wolves. Social groups, especially hunter identity, have been shown to be strong predictors of attitudes towards wolves and other large carnivores [19,55,57,59,60]. For example, in a meta-analysis of attitudes toward wolves and bears across Europe, Dressel et al. [61] found that farmers and hunters were less likely to hold positive views toward wolves and bears compared to the general public. Hunter identity was a significant predictor of favorability towards wolves in a Wisconsin state-wide survey [19]. Livestock ownership was not a significant predictor of anticipated changes in tolerance, even though others have found it to be a significant predictor of favorability towards wolves [19,61,62]. Rather, we found a significant and consistent positive effect on anticipated changes in attitudes for those who reported to have experience with loss of livestock to wildlife. Likewise, researchers studying attitudes towards large carnivores in Wisconsin and Norway found that people who had lost livestock to carnivores were less tolerant or had more negative attitudes towards carnivores [59,63]. Puri et al. [33] found that tolerance of urban wildlife was partially influenced by prior interactions, particularly peoples’ beliefs regarding whether they can mitigate conflict with wildlife or not. Interestingly, our survey asked individuals to identify if they had experience with the loss of livestock to any species of wildlife, not specifically wolves. Loss of livestock to any species of wildlife likely influences perceived risks regarding *any* potential predatory species of wildlife and appears to result in a general preference for lethal control opportunities. Efforts to mitigate and prevent any type of human–wildlife conflict may, in turn, lead to greater tolerance of all wildlife, particularly for large carnivores.

### 4.2. The Empowerment Hypothesis Explored

Our results suggest policy changes allowing for more state management authority, including the ability to implement legal lethal removal (lethal control or legal hunting or trapping), particularly in conflict situations, *could* result in increased tolerance towards wolves for those that are currently ambivalent or negative towards wolves. These findings generally support the empowerment hypothesis, as described by USDA APHIS WS et al. [22], that, “*…Risks considered involuntary by an individual are less likely to be viewed as acceptable whereas risks that can be controlled are generally considered to be more acceptable… a government which simultaneously imposes the risk of wolf depredation (i.e., supports wolf recovery) and prohibits individuals from effectively reducing those risks (i.e., no chance for removal of problem wolves) is creating an intolerance of the wolf presence…*” (ref. [22], pp. 26–27)

Thus, individuals living alongside large carnivores in more rural settings may view wolf presence as an imposed risk and may become intolerant of wolves if they perceive that they have limited say over how the species is managed to reduce risks. Similar disenfranchisement of local communities inspired recent growth in community-based conservation, i.e., empowering and engaging local people in conservation appears to be critical to conservation success [1,3,7,10]. Such situations are not limited to wolves alone. For example, Raycraft [14] described a situation in Tanzania where the socioeconomic impacts of conflict with spotted hyenas (*Crocuta corcuta)* with few options for resolution (due to socioeconomic and policy reasons) resulted in a general intolerance of the species. As summarized by Raycraft [14], “The result of these socioeconomic impacts is that local pastoralists *despise* spotted hyenas and most would like to see them completely eradicated” (p. 110). Raycraft [14] also provided evidence of retaliatory killing of spotted hyenas. 

The empowerment hypothesis also infers that we can expect tolerance towards wolves to improve for individuals, particularly those who are most locally impacted by wolves if policy decisions more closely align with their preferred management approaches. Bradshaw et al. [19] found that landowners, hunters/trappers, farmers/livestock producers, and individuals living in wolf range were significantly more likely to experience feelings of anger and frustration when thinking about wolves. Such feelings could easily influence feelings of tolerance. Browne-Nunez et al. [17] reported that social groups that are generally more intolerant towards wolves (deer hunters, bear hunters, livestock producers who have lost livestock to wolves) expressed frustration and a lack of empowerment to deal with wolves causing conflict. The work of Browne-Nunez et al. [17] suggests that feelings of frustration and anger are often focused on wolf policy rather than wolves themselves. Based on our findings, individuals from such backgrounds (i.e., hunters and those with human–wildlife conflict experience, living in wolf range) were more likely to anticipate increases in tolerance following changes in wolf policies. Our results add to a growing body of literature demonstrating the linkages between wolf policy and tolerance of wolves [8,11,17,19,44].

### 4.3. Nuance Associated with the Empowerment Hypothesis

While our work supports the empowerment hypothesis in general [22], there is an important nuance to consider during policy decision-making, particularly as it relates to the specifics of the legal killing of wolves for management purposes. For example, lethal removal of wolves in response to conflict situations was clearly perceived differently by tolerance clusters than regulated hunting and trapping seasons (Figure 4 and Figure 5). There was also clear *variation* within tolerance clusters as it relates to behavioral intentions and policy preferences (Figure 3 and Figure 4). Thus, the nuances of policy preferences remain important, i.e., details and justification of wolf killing appear to be critically important considerations for many when evaluating any such policy. For example, Bradshaw et al. [19] received responses from 3158 Wisconsin residents and found that the majority support lethal control of wolves causing conflict in both 2014 and 2022, regardless of conflict type (they examined attitudes towards four common types of conflict) or whether respondents were in wolf range or not. They also found lethal control via wildlife professionals or landowner permits was the most acceptable response to wolf–human conflict in Wisconsin in both 2014 and 2022. Yet, since 2014, except for part of 2021, lethal control and landowner permits were not possible, except for rare cases of human safety concern, due to wolves in Wisconsin being federally listed as endangered. Thus, wolf policy that prevented lethal control authority from killing wolves involved in conflicts was largely outside of the policy preferences of most Wisconsin residents (e.g., refs. [11,19]), particularly those that were ambivalent or held negative views toward wolves. Such a scenario could further enhance frustrations associated with wolf management and potentially lead to a decrease in tolerance for wolves among these groups if policy remains unchanged into the future. 

Contrary to what one would expect based on the empowerment hypothesis, Browne-Nunez et al. [17] and Hogberg et al. [18] found that attitudes towards wolves in Wisconsin did not improve after a period with increased legal lethal removal of wolves. In fact, ref. [18] reported a decrease in attitudes towards wolves. What could potentially explain these seemingly contradictory findings? Bradshaw et al. [19] found attitudes towards wolves became more favorable between 2014 and 2022 in Wisconsin, both within wolf range and outside of wolf range. Yet, wolf policies during this period were variable and surveys were completed during a period with wolf harvest (2014) and immediately following a period of delisting and relisting (2022). Thus, the longitudinal relationship between attitudes and policy may be difficult to interpret without annual survey efforts over an extended period. 

Additionally, one might ask, “How fast can we expect tolerance to change?” Browne-Nunez et al. [17] completed their work while Wisconsin’s first-ever regulated wolf hunting and trapping season was still on-going and [18] completed their work less than one year after the first harvest. According to the cognitive hierarchy, we know that behaviors are more flexible and quicker to change, and attitudes are more rigid and slower to change [64]. Thus, it is unlikely that changes in attitudes can be expected over short periods of time. Behaviors and behavioral intentions are expected to change more quickly. Observations of a decrease in issuance (121, 60, and 47 permits issued, respectively) and use (fourteen, eight, and two permits with wolves killed, respectively) of landowner permits for those experiencing conflicts with wolves to be able to kill wolves on their land from 2012 to 2014 (WDNR data, R. Johnson, *pers. comm*.) may be suggestive of slow progress in tolerance for individuals most likely to anticipate increases in tolerance following the implementation of such policies. Reductions in the illegal killing of wolves when certain types of legal killing of wolves are sanctioned may also be suggestive of improving tolerance towards wolves [11,43,45], but recent scientific debate makes these findings less clear [31,46,47,50,51,52].

Similarly, Olson et al. [11] highlight an additional caveat for those testing the empowerment hypothesis to consider, that inconsistency in policy, i.e., pendulum swings in wolf management due to win–lose conservation conflict, actually led to decreases in tolerance towards wolves. In other words, attitudes are shaped not only by current policy but also by past changes in policy and the stability of policies over time. Wolf killing in Wisconsin from 2003 to 2015, transitioned from federally endangered to lethal control via wildlife professionals to the inclusion of landowner permits to a legislatively mandated wolf hunt. However, this “transition” took twelve years, represents a series of mostly independent efforts from different entities of government (i.e., not an organized concerted effort by a single entity), was marked by regular periods of full federal protection for wolves, and was temporary (e.g., wolves were relisted as federally endangered in 2015 until 2020). This “transition” was not a smooth linear progress as some scholars have suggested (e.g., “wolf killing progressively liberalized”; p. 2, ref. [65]). In fact, wolf policy in Wisconsin since 2003 has oscillated as a result of intense sociopolitical conflict [11,54]. We suspect that lack of stability in wolf policy and history with past policy changes may provoke stronger feelings of anger and frustration than policies that are simply incompatible with stakeholder preferences [8,10,44,66]. Moreover, if attitudes are slower to change, we presume frequent policy changes may make it difficult to detect actual changes in attitude because those changes would require time and with that time consistency of policy [7]. Thus, while our work suggests that policy changes may improve tolerance of some groups, constancy of such policy changes and the process through which policies are set may determine the strength and durability of shifts in tolerance. We encourage future scholars to further explore how (in)stability of policies over time shapes tolerance towards wildlife or satisfaction with wildlife conservation policies. We also encourage future scholars to directly assess the rate of change for attitudes, behavioral intentions, and behaviors as a result of policy changes at different time scales. Therefore, while our work supports the empowerment hypothesis in general [22], there is important nuance to consider during policy decision-making, particularly as it relates to the specifics of legal killing of wolves for management purposes. 

## 5. Conclusions

Redpath et al. [10] argued that if stakeholders are unable to find common ground and see the challenge of wildlife conservation as a shared one, then they will fall into a cyclic and intense sociopolitical win–lose game of conservation conflict [7,8,11,66]. Our results suggest that certain wolf policies may provide non-zero-sum outcomes that Redpath et al. [10] encourage. Policies with some shared support amongst tolerance clusters were reclassifying wolves as threatened under the Endangered Species Act or delisting wolves from the Endangered Species Act (Figure 3, Figure 4 and Figure 5). Notably, the wording of our questions and current state and federal policies help highlight nuanced areas of agreement. For example, there was less agreement about a regulated hunting and trapping season (Figure 3 and Figure 4), even though when wolves are delisted in Wisconsin, state law requires the state to hold a hunting and trapping season [54]. There was less support to remove federal protections for wolves than there was to give state management authority to kill wolves attacking livestock or pets near homes, yet such authority is granted when federal protections are removed. Thus, we infer that wolf policies that give the state management authority to kill wolves involved in conflict scenarios (i.e., lethal control) is an area of potential agreement amongst all tolerance clusters and could result in increased tolerance towards wolves, especially for individuals who already view wolves ambivalently or negatively [57]. Based on our results, wolf policies (in Wisconsin) that lean more protectionist or that include wolf hunting and trapping, must be done conservatively if the goal is to establish policy decisions that accommodate stakeholder’s perspectives and potentially reduce sociopolitical conflict over management decision-making. However, we acknowledge that such a goal may not be a priority in all conservation decision-making.

Protections under the Endangered Species Act were integral for wolves to recolonize and repopulate a large area of suitable habitat in the Upper Great Lakes Region. The type of protection offered by the Endangered Species Act was paramount during initial recovery efforts and is a critically important tool in the conservation toolbox. However, in cases where large carnivore populations are robust enough to shift from recovery to maintenance, long-term coexistence between large carnivores and people who live in close proximity to those carnivores may be heavily influenced by tolerance [1]. Our results support the empowerment hypothesis of tolerance, suggesting that individuals who are more intolerant of large carnivores may become more tolerant of large carnivores when carnivore policies better align with the perspectives of locals living alongside those large carnivores. Likewise, adapting policies to maximize tolerance across stakeholder groups may be beneficial for long-term conservation of large carnivores [3].

Large carnivore conservationists must balance conservation objectives with the preferences of local people living with large carnivores. They must also balance both short- and long-term conservation priorities. The fulcrum of this balance may shift over time as local preferences or the status of a species change. Thus, monitoring local policy preferences and tolerance of large carnivores may be just as important as carnivore population monitoring. 

## Figures and Tables

**Table 1:** Generalized logistic regression model parameter estimates (±95% confidence interval) for the top models predicting anticipated changes in tolerance towards wolves in response to three realistic policy change scenarios: “My tolerance for Wisconsin wolves would increase if…”: (a) “…they were **not** federally protected”, (b) “…the state had management authority to kill wolves attacking livestock or pets near homes”, (c) “…there was a regulated hunting and trapping season for wolves”, for northern Wisconsin, USA, 2015 (*n* = 212). The two highest-ranked models for all three scenarios contained the variables hunter identity (*Hunter*), experience with loss of livestock to a predator (*Lost livestock*), and tolerance cluster assignment (*Positive*, *Ambivalent*, or *Negative*) (Supplemental Material). We included both the first ^1^ and second ^2^ top-ranked models for the scenario “…under state management authority…” for ease of comparison.

Scenario	Intercept (*Positive* *)	*Hunter*	*Lost* *Livestock*	* *Ambivalent*	* *Negative*
(a) *…were **not** federally protected…*	**1.62 (0.25)**	**0.29 (0.29)**	**0.31 (0.31)**	**0.98 (0.31)**	**1.38 (0.41)**
(b) *…under state authority…* ^1^	**2.63 (0.27)**	NA	**0.36 (0.31)**	**0.81 (0.33)**	**1.27 (0.45)**
(b) *…under state authority…* ^2^	**2.60 (0.29)**	0.11 (0.33)	0.32 (0.33)	**0.81 (0.33)**	**1.25 (0.45)**
(c) *…regulated hunting…*	**1.57 (0.27)**	**0.43 (0.31)**	0.30 (0.31)	**1.09 (0.31)**	**1.85 (0.43)**

Bold indicates coefficients with 95% confidence intervals that do not overlap 0. * Tolerance cluster assignment intercept adjustment; cluster represents respondents initial tolerance towards wolves at the time of the survey; intercept represents individuals that already have a positive tolerance towards wolves.

## Data Availability

The data presented in this study are available on request from the corresponding author.

## Supplementary Materials

The following supporting information can be downloaded at: https://www.mdpi.com/article/10.3390/ani14162358/s1, Table S1: Generalized linear regression model ranking for self-reported, anticipated changes in tolerance for wolves if they were not federally protected according to survey respondents from northern Wisconsin, 2015 (*n* = 212). Candidate models included the following variables: educational background (*Education*; categorical), gender (categorical), age (quantitative), hunter identity (*Hunter*; categorical), and tolerance assignment cluster (i.e., initial tolerance towards wolves; *Cluster*; categorical); Table S2: Generalized logistic regression model ranking of self-reported, anticipated change in tolerance for wolves under state authority according to survey respondents from northern Wisconsin, 2015 (*n* = 212). Candidate models included the following variables: educational background (*Education*; categorical), gender (categorical), age (quantitative), hunter identity (*Hunter*; categorical), and tolerance assignment cluster (i.e., initial tolerance towards wolves; *Cluster*; categorical); Table S3: Generalized logistic regression model ranking of self-reported, anticipated change in tolerance toward wolves if there was regulated hunting according to survey respondents from northern Wisconsin, 2015 (*n* = 212). Candidate models included the following variables: educational background (*Education*; categorical), gender (categorical), age (quantitative), hunter identity (*Hunter*; categorical), and tolerance assignment cluster (i.e., initial tolerance towards wolves; *Cluster*; categorical).
