# Supplementary material for: Changing the Tolerance of the Intolerant: Does Large Carnivore Policy Matter?
Source: Animals (Basel). 2024 Aug 15;14(16):2358. doi: 10.3390/ani14162358 (PMC11350789; doi:10.3390/ani14162358)
Supplement: Supplementary file 1 [file animals-14-02358-s001.zip › animals-3110497-supplementary.pdf]

SUPPLEMENTAL MATERIAL

Table S1. Generalized linear regression model ranking for self-reported, anticipated changes in tolerance for wolves if they were **not** federally protected according to survey respondents from northern Wisconsin, 2015 (n=212). Candidate models included the following variables: educational background (*Education*; categorical), gender (categorical), age (quantitative), hunter identity (*Hunter*; categorical), and tolerance assignment cluster (i.e., initial tolerance towards wolves; *Cluster*; categorical).

Model	K	AIC_c	ΔAIC_c	ω_i
Hunter + Lost livestock + Cluster	5	605.75	0.00	0.46
Lost livestock + Cluster	4	607.39	1.64	0.20
Hunter + Cluster	4	607.54	1.79	0.19
Hunter + Own livestock + Cluster	5	609.42	3.67	0.07
Hunter * Lost livestock * Cluster	12	611.02	5.27	0.03
Hunter * Own livestock * Cluster	12	612.06	6.31	0.02
Cluster	3	612.51	6.76	0.02
Own livestock + Cluster	4	613.83	8.08	0.01
Hunter * Age * Lost livestock	8	643.67	37.92	0.00
Hunter + Age + Lost livestock	4	650.99	45.24	0.00
Hunter + Lost livestock	3	653.63	47.88	0.00
Hunter + Gender + Lost livestock	4	655.70	49.95	0.00
Lost Livestock	2	657.05	51.30	0.00
Hunter * Gender * Lost livestock	8	662.47	56.73	0.00
Age	2	674.57	68.82	0.00
Gender + Age	3	675.52	69.77	0.00
Education	2	676.22	70.47	0.00
Null	1	676.31	70.56	0.00
Own livestock	2	676.35	70.60	0.00
Gender	2	676.49	70.74	0.00

ΔAIC_c = difference between model second-order Akaike information criterion (AIC_c) and lowest AIC_c in the model set. ω = Akaike model weight. K = number of estimable parameters.

Table S2. Generalized logistic regression model ranking of self-reported, anticipated change in tolerance for wolves under state authority according to survey respondents from northern Wisconsin, 2015 (n=212). Candidate models included the following variables: educational background (*Education*; categorical), gender (categorical), age (quantitative), hunter identity (*Hunter*; categorical), and tolerance assignment cluster (i.e., initial tolerance towards wolves; *Cluster*; categorical).

Model	K	AIC_c	ΔAIC_c	ω_i
Lost livestock + Cluster	4	644.11	0.00	0.45
Hunter + Lost livestock + Cluster Cluster	5	645.79	1.68	0.19
Cluster	3	646.82	2.72	0.12
Hunter + Cluster	4	647.21	3.10	0.10
Own livestock + Cluster	4	647.43	3.31	0.09
Hunter + Own livestock + Cluster	5	648.25	4.14	0.06
Hunter * Lost livestock * Cluster	12	653.95	9.85	0.00
Hunter * Own livestock * Cluster	12	659.15	15.04	0.00
Hunter + Age + Lost livestock	4	660.27	16.16	0.00
Hunter * Age * Lost livestock	8	663.87	19.76	0.00
Lost Livestock	2	674.83	30.72	0.00
Age	2	674.96	30.85	0.00
Hunter + Lost livestock	3	675.50	31.39	0.00
Gender + Age	3	676.52	32.41	0.00
Hunter + Gender + Lost livestock	4	677.28	33.17	0.00
Hunter * Gender * Lost livestock	8	684.39	40.28	0.00

Own livestock	2	687.75	43.64	0.00
Null	1	688.61	44.50	0.00
Gender	2	688.83	44.72	0.00
Education	2	690.50	46.39	0.00

ΔAIC_c = difference between model second-order Akaike information criterion (AIC_c) and lowest AIC_c in the model set. ω = Akaike model weight. *K* = number of estimable parameters.

Table S3. Generalized logistic regression model ranking of self-reported, anticipated change in tolerance toward wolves if there was regulated hunting according to survey respondents from northern Wisconsin, 2015 (n=212). Candidate models included the following variables: educational background (*Education*; categorical), gender (categorical), age (quantitative), hunter identity (*Hunter*; categorical), and tolerance assignment cluster (i.e., initial tolerance towards wolves; *Cluster*; categorical).

**Model**	**K**	**AIC_c**	**Δ AIC_c**	**ω_i**
Hunter + Lost livestock + Cluster	5	621.86	0.00	0.46
Hunter + Cluster	4	623.09	1.22	0.25
Hunter + Own livestock + Cluster	5	623.15	1.29	0.24
Lost livestock + Cluster	4	627.29	5.43	0.03
Hunter * Lost livestock * Cluster	12	629.74	7.88	0.01
Hunter * Own livestock * Cluster	12	631.72	9.86	0.00
Cluster	3	632.87	11.00	0.00
Own livestock + Cluster	4	634.24	12.38	0.00
Hunter + Age + Lost livestock	4	684.79	62.93	0.00
Hunter + Lost livestock	3	689.31	67.45	0.00
Hunter * Age * Lost livestock	8	689.38	67.52	0.00
Hunter + Gender + Lost livestock	4	691.28	69.42	0.00
Hunter * Gender * Lost livestock	8	696.10	74.23	0.00
Lost Livestock	2	697.49	75.63	0.00
Age	2	718.96	97.10	0.00
Education	2	720.02	98.15	0.00
Gender + Age	3	720.35	98.48	0.00
Null	1	721.79	99.92	0.00
Gender	2	722.43	100.57	0.00
Own livestock	2	723.78	100.92	0.00

ΔAIC_c = difference between model second-order Akaike information criterion (AIC_c) and lowest AIC_c in the model set. ω = Akaike model weight. *K* = number of estimable parameter.
